# The Calcium-Sensing Receptor Mediates Bone Turnover Induced by Dietary Calcium and Parathyroid Hormone in Neonates

**DOI:** 10.1002/jbmr.300

**Published:** 2010-11-23

**Authors:** Lei Shu, Ji Ji, Qi Zhu, Guofan Cao, Andrew Karaplis, Martin R Pollak, Edward Brown, David Goltzman, Dengshun Miao

**Affiliations:** 1 Laboratory of Reproductive Medicine, Research Center for Bone and Stem Cells, Nanjing Medical UniversityNanjing, Jiangsu, People's Republic of China; 2 Department of Medicine, McGill UniversityMontreal, Quebec, Canada; 3 Division of Endocrinology, Diabetes and Hypertension, Department of Medicine, Brigham and Women's Hospital, Harvard Medical SchoolBoston, MA USA; 4 Department of Geriatrics, First Affiliated Hospital of Nanjing Medical UniversityNanjing, Jiangsu, People's Republic of China

**Keywords:** CALCIUM-SENSING RECEPTOR, PARATHYROID HORMONE, DIETARY CALCIUM, BONE TURNOVER

## Abstract

We have investigated, in neonates, whether the calcium-sensing receptor (CaR) mediates the effects of dietary calcium on bone turnover and/or modulates parathyroid hormone (PTH)–induced bone turnover. Wild-type (WT) pups and pups with targeted deletion of the *Pth* (*Pth*^–/–^) gene or of both *Pth* and *CaR* (*Pth*^–/–^*CaR*^–/–^) genes were nursed by dams on a normal or high-calcium diet. Pups nursed by dams on a normal diet received daily injections of vehicle or of PTH(1–34) (80 µg/kg) for 2 weeks starting from 1 week of age. In pups receiving vehicle and fed by dams on a normal diet, trabecular bone volume, osteoblast number, type 1 collagen–positive area, and mineral apposition rate, as well as the expression of bone-formation-related genes, all were reduced significantly in *Pth*^–/–^ pups compared with WT pups and were decreased even more dramatically in *Pth*^–/–^*CaR*^–/–^ pups. These parameters were increased in WT and *Pth*^–/–^ pups but not in *Pth*^–/–^*CaR*^–/–^ pups fed by dams on a high-calcium diet compared with pups fed by dams on a normal diet. These parameters also were increased in WT, *Pth*^–/–^, and *Pth*^–/–^*CaR*^–/–^ pups following exogenous PTH treatment; however, the percentage increase was less in *Pth*^–/–^*CaR*^–/–^ pups than in WT and *Pth*^–/–^ pups. In vehicle-treated pups fed by dams on either the normal or high-calcium diet and in PTH-treated pups fed by dams on a normal diet, the number and surfaces of osteoclasts and the ratio of RANKL/OPG were reduced significantly in *Pth*^–/–^ pups and less significantly in *Pth*^–/–^*CaR*^–/–^ pups compared with WT pups. These parameters were further reduced significantly in WT and *Pth*^–/–^ pups from dams fed a high-calcium diet but did not decrease significantly in similarly treated *Pth*^–/–^*CaR*^–/–^ pups, and they increased significantly in PTH-treated pups compared with vehicle-treated, genotype-matched pups fed by dams on the normal diet. These results indicate that in neonates, the CaR mediates alterations in bone turnover in response to changes in dietary calcium and modulates PTH-stimulated bone turnover. © 2011 American Society for Bone and Mineral Research.

## Introduction

The calcium-sensing receptor (CaR) plays a central role in controlling systemic calcium homeostasis, predominately through its effects on regulation of parathyroid hormone (PTH) secretion by the parathyroid glands and urinary calcium excretion by the kidney.(1–3) Recent evidence clearly has shown that the CaR is expressed and modulates cell function in a variety of bone cells, including osteoblasts,(4–8) stromal cells,(9) osteoclasts,(6,10,11) and chondrocytes.(4) Previous studies on cultured osteoblasts have shown that CaR activation not only stimulates the proliferation of these cells but also promotes their differentiation and mineralization. Cellular proliferation and expression of osteoblast differentiation markers, including *Cbfa-*1, *alkaline phosphatase* (*Alp*), *type 1 collagen*, and *osteocalcin*, and mineralized nodule formation were increased significantly in rat calvarial osteoblasts treated with high calcium. Inhibition of the CaR by NPS 89636 (a CaR antagonist) blocked responses in vitro to CaR agonists, suggesting that high-calcium-induced osteoblastic differentiation was mediated by the CaR.(5) Abolition of CaR function by stably transfecting MC3T3-E1 cells with either a CaR antisense vector or a vector containing the dominant-negative R185Q variant of the CaR resulted in diminishing ALP activity, osteocalcin expression, and mineralization in mouse osteoblastic cells.(12) More recently, mice with conditional knockout of the CaR in early committed osteoblasts were generated by breeding a novel mouse line carrying a *CaR* gene allele in which exon 7 had been floxed with transgenic mice expressing Cre recombinase under the control of a 2.3-kb type 1 collagen promoter.(13) Smaller, undermineralized skeletons with significant reductions in bone volume and bone mineral density (BMD) were observed in the femur and vertebrae from the conditional knockout mice. This study demonstrated a critical role for the CaR at early stages of osteoblastic differentiation and bone development potentially by promoting osteoblast maturation.

Previous studies failed to find a direct role for CaR in mice homozygous for targeted disruption of exon 5 of the *CaR* gene, which encodes a segment of the extracellular domain of this receptor.(14,15) These observations may have been confounded by the severe hyperparathyroidism and accompanying hypercalcemia and hypophosphatemia in this animal model. To better understand the direct effects of the CaR on bone and cartilage function, correction of hyperparathyroidism is required in this CaR-deficient mouse model. Therefore, a double-knockout model was established by ablating the *Pth* gene in the CaR-deficient mice(16,17) to correct the severe hyperparathyroidism, hypercalcemia, and hypophosphatemia observed in the CaR-deficient mice. The results of this study showed that elimination of hyperparathyroidism rescued the mice from the increased neonatal mortality as well as the rickets-like skeletal abnormality in the *CaR*^–/–^ mice. However, any essential, nonredundant role for CaR in regulating chondrogenesis or osteogenesis was not identified based on analysis of the skeleton of this adult double-knockout model. It is unclear why there are discrepancies between the findings from the mice with the osteoblastic-specific knockout of exon 7 of the *CaR* gene(13) and those from mice with conventional global knockout of exon 5 of both the *CaR* and *Pth* genes(14,16) regarding the physiologic significance of the CaR in osteoblasts. It is possible, however, that the alternatively spliced *CaR* lacking exon 5 that is expressed in the global exon 5 knockout mice may compensate to some extent in bone for loss of the full-length *CaR*.

PTH can stimulate bone formation in adult and aged animals of either sex and in animals with osteopenia induced by disuse, denervation, and immobilization.(18) The anabolic action of PTH on bone has been validated in humans(19–21) and its antifracture efficacy established in postmenopausal osteoporotic women.(22) At present, however, the physiologic basis of PTH anabolism remains unclear.(23,24) A large body of evidence demonstrates that sufficient calcium intake augments bone gain during growth, retards age-related bone loss, and reduces osteoporotic fracture risk,(25–27) but the mechanisms underlying the positive effect of increased calcium intake on bone mass are unknown. Our recent study indicates that the skeletal anabolic action of PTH in neonates results not only from its direct action via the PTH receptor to increase the osteoblast pool but also by an indirect action mediated through PTH-induced increases in the extracellular calcium concentration.(28) However, it is unclear whether the increase in extracellular calcium caused by PTH administration acts cooperatively with a skeletal anabolic action mediated via the CaR.

In this study, we attempted to determine whether deletion of *CaR* in PTH-deficient mice (1) leads to a skeletal phenotype distinct from that caused by deletion of *Pth* alone before weaning, (2) abolishes skeletal responses to dietary calcium supplementation, and (3) reduces skeletal responses to exogenous PTH administration. To explore these issues, we examined pups from wild-type (WT), *Pth*^–/–^, and *Pth*^–/–^*CaR*^–/–^ mice nursed by dams on a normal calcium diet or on a high-calcium diet. In addition, we treated some pups nursed by dams on a normal calcium diet with daily injections of vehicle or of PTH(1–34) for 2 weeks starting from 1 week of age and assessed skeletal homeostasis.

## Materials and Methods

### Derivation of *Pth*^–/–^ and *Pth*^–/–^*CaR*^–/–^ mice

The derivation of the two parental strains of *Pth*^–/–^ mice and *CaR*^–/–^ mice by homologous recombination in embryonic stem cells was described previously by Miao and colleagues(29) and Ho and colleagues,(15) respectively. *Pth*^+/–^ mice were bred with *CaR*^+/–^ mice to generate *Pth*^+/–^*CaR*^+/–^ mice. *Pth*^+/–^*CaR*^+/–^ mice were bred among themselves to generate *Pth*^–/–^*CaR*^–/–^ pups. These mice were maintained on a mixed genetic background with contributions from C57B6, 129/SvJ, and 129/SvEv strains. The genotypes of the animals were determined by polymerase chain reaction (PCR).

### In vivo experiments

All animal experiments were approval by the Institutional Animal Care and Use Committee. Mice were maintained in a virus- and parasite-free barrier facility and exposed to a 12-hour light/dark cycle. To determine whether PTH and calcium interact in neonatal calcium and skeletal homeostasis, WT, *Pth*^–/–^, or *Pth*^+/–^*CaR*^+/–^ dams were fed with rodent diets from Harlan Teklad (Madison, WI, USA) containing either normal levels of calcium and phosphate (1% Ca, 0.6% phosphate) or high-calcium and moderate-phosphate levels (2% Ca and 0.4% phosphate). Serum biochemistry was not significantly different in the *Pth*^+/–^*CaR*^+/–^ mice than in the WT mice. Dietary phosphate content in the high-calcium diet was reduced moderately to decrease phosphate absorption and resulting hyperphosphatemia and to facilitate increasing the blood calcium concentration in the hypoparathyroid *Pth*^–/–^ and *Pth*^–/–^*CaR*^–/–^ mice. The high-calcium, moderate-phosphate diet was found to normalize hypocalcemia and hyperphosphatemia in *Pth*^–/–^ and *Pth*^–/–^*CaR*^–/–^ mice and is referred to as the *high-calcium diet*. Litter size was equalized to 5 to 6 pups per dam to equalize suckling intensity. On day 7 postpartum, pups nursed by dams on the normal calcium diet received daily injections of vehicle or of PTH(1–34) (80 µg/kg) for 2 weeks. At the end of experiments, milk of dams and blood of pups were collected and used for biochemical analysis, and long bones of pups then were removed for the analyses described below.

### Biochemistry and hormone analyses

Milk calcium (milk was diluted 1:100 in distilled water) was measured with an atomic absorptiometer as described previously.(30) Serum calcium and phosphorus levels were determined by an autoanalyzer (Beckman Synchron 67; Beckman Coulter, Inc., Fullerton, CA, USA). Urine calcium levels were determined using the *o*-cresolphthalein-complexone method (Sigma-Aldrich, St Louis, MO, USA), urine creatinine was measured by the colorimetric alkaline picrate method (Sigma Kit 555, Sigma-Aldrich), and urine calcium/creatinine ratio was calculated. Milk and serum 1,25-dihydroxyvitamin D_3_ [1,25(OH)_2_D_3_] was measured by RIA (Immuno-Diagnostic Systems, Bolden, UK), and PTH-related protein (PTHrP) was measured by a two-site immunoradiometric assay (Immutopics, San Clemente, CA, USA). Serum PTH was measured using an ELISA (Immutopics).

### Radiography and measurement of BMD

For radiography, tibias were removed and dissected free of soft tissue, and X-ray images were taken with a Faxitron machine (Model 805; Faxitron X-Ray Corp., Wheeling, IL, USA) under constant conditions (22 kV, 4-minute exposure) using Kodak X-Omat TL film (Eastman Kodak Co., Rochester, NY, USA). For measurement of tibial BMD, a PIXImus densitometer (Lunar PIXImus Corp., Madison, WI, USA) was used (5-minute image acquisition with the precision of 1% coefficient of variation for skeletal BMD). The PIXImus software automatically calculated the BMD and recorded the data in Microsoft Excel files (Microsoft Corp., Redmond, WA, USA).

### Micro–computed tomography (µCT)

Tibias obtained from 3-week-old mice were dissected free of soft tissue, fixed overnight in 70% ethanol, and analyzed by µCT with a SkyScan 1072 scanner and associated analysis software (SkyScan, Antwerp, Belgium) as described previously.(31) Briefly, image acquisition was performed at 100 kV and 98 µA with a 0.9-degree rotation between frames. During scanning, the samples were enclosed in tightly fitting plastic wrap to prevent movement and dehydration. Thresholding was applied to the images to segment the bone from the background. 2D images were used to generate 3D renderings using the 3D Creator software supplied with the instrument (SkyScalciumn). The resolution of the µCT images is 18.2 µm.

### Quantitative real-time RT-PCR

Reverse-transcription (RT) reactions were performed using the SuperScript First-Strand Synthesis System (Invitrogen, Carlsbad, CA, USA) as described previously.(32) Real-time polymerase chain reaction (PCR) was performed using a LightCycler system (Roche Molecular Biochemicals, Indianapolis, IN, USA). The conditions were 2 µL of LightCycler DNA Master SYBR Green I (Roche), 0.25 µM of each 5' and 3' primer (Table 1), and 2 µL of sample and/or H_2_O to a final volume of 20 µL. Samples were amplified for 35 cycles with a temperature transition rate of 20°C/s for all three steps, which were denaturation at 94°C for 10 seconds, annealing for 5 seconds, and extension at 72°C for 20 seconds. SYBR green fluorescence was measured to determine the amount of double-stranded DNA. To discriminate specific from nonspecific cDNA products, a melting curve was obtained at the end of each run. Products were denatured at 94°C for 30 seconds; the temperature then was decreased to 55°C for 15 seconds and raised slowly from 55 to 94°C using a temperature transition rate of 0.18°C/s. To determine the number of copies of target DNA in the samples, purified PCR fragments of known concentration were serially diluted and served as external standards for each experiment. Data were normalized to *GAPDH* levels.

**Table 1:** Real-Time RT–PCR Primers Used with Their Name, Orientation (S = Sense; AS = Antisense), Sequence, Annealing Temperature (Tm), and Length of Amplicon (bp)

Name	S/AS	Sequence	Tm(°C)	bp
*Cbfa*-1	S	GTGACACCGTGTCAGCAAAG	55	356
	AS	GGAGCACAGGAAGTTGGGAC		
*Rankl*	S	GGTCGGGCAATTCTGAATT	57	813
	AS	GGGGAATTACAAAGTGCACCAG		
*Opg*	S	TGGAGATCGAATTCTGCTTG	57	719
	AS	TCAAGTGCTTGAGGGCATAC		
*Alp*	S	CTTGCTGGTGGAAGGAGGCAGG	55	393
	AS	GGAGCACAGGAAGTTGGGAC		
*type 1 collagen*	S	TCTCCACTCTTCTAGTTCCT	55	269
	AS	TTGGGTCATTTCCACATGC		
*osteocalcin*	S	CAAGTCCCACACAGCAGCTT	55	370
	AS	AAAGCCGAGCTGCCAGAGTT		
*GAPDH*	S	GGTCGGTGTGAACGGATTTG	55	508
	AS	ATGAGCCCTTCCACAATG		

### Histology

Tibias were removed and fixed in 2% paraformaldehyde containing 0.075 M lysine and 0.01 M sodium periodate overnight at 4°C and processed as described previously.(33) Proximal ends of tibias were decalcified in EDTA-glycerol solution for 5 to 7 days at 4°C. Decalcified right tibias were dehydrated and embedded in paraffin, after which 5-µm sections were cut on a rotary microtome. The sections were stained with hematoxylin and eosin (H&E) or histochemically for tartrate-resistant acid phosphatase (TRACP) activity or immunohistochemically, as described below. For calcein labeling, mice were injected intraperitoneally with 10 µg calcein per gram of body weight (C-0875; Sigma) at 8 and 3 days prior to killing, as described previously.(34) Left tibiaa were embedded in LR white acrylic resin (London Resin Co., Ltd., London, UK), and 1-µm sections were viewed and imaged using fluorescence microscopy. The double-calcein-labeled widths of trabeculae were measured in the secondary spongiosa of the proximal ends of tibias using Northern Eclipse image-analysis software Version 6.0 (Empix Imaging, Inc., Mississauga, Ontario, Canada), and the mineral apposition rate (MAR) was calculated as the interlabel width/labeling period. TRACP staining was performed using a modification of a previously described protocol.(32) In brief, dewaxed sections were preincubated for 20 minutes in buffer containing 50 mM sodium acetate and 40 mM sodium tartrate at pH 5.0. Sections then were incubated for 15 minutes at room temperature in the same buffer containing 2.5 mg/mL of naphthol AS-MX phosphate (Sigma) in dimethylformamide as substrate and 0.5 mg/mL of fast garnet GBC (Sigma) as a color indicator for the reaction product. After washing with distilled water, the sections were counterstained with methyl green and mounted in Kaiser's glycerol jelly.

### Immunohistochemical staining

Immunohistochemical staining for type 1 collagen was performed using affinity-purified goat anti-mouse type 1 collagen antibody (Southern Biotechnology Associates, Birmingham, AL, USA) as described previously.(33) Briefly, dewaxed and rehydrated paraffin-embedded sections were incubated with methanol–hydrogen peroxide (1:10) to block endogenous peroxidase activity and then washed in Tris-buffered saline (pH 7.6). The slides then were incubated with the primary antibody overnight at room temperature. After rinsing with Tris-buffered saline for 15 minutes, tissues were incubated with secondary antibody (biotinylated goat anti-rabbit IgG or biotinylated goat anti-mouse IgG; Sigma). Sections then were washed and incubated with the Vectastain Elite ABC reagent (Vector Laboratories, Burlington, Ontario, Canada) for 45 minutes. Staining was developed using 3,3-diaminobenzidine (2.5 mg/mL) followed by counterstaining with Mayer's hematoxylin.

### Bone marrow cell cultures

Tibias and femurs of 3-week-old WT, *Pth*^–/–^, and *Pth*^–/–^*CaR*^–/–^ pubs fed by dams on the normal diet or the high-calcium diet that were administrated the exogenous PTH(1–34) were removed under aseptic conditions, and bone marrow cells were flushed out with DMEM containing 10% fetal calf serum (FCS), 50 µg/mL of ascorbic acid, 10 mM β-glycerophosphate, and 10^−8^ M dexamethasone. Cells were dispersed by repeated pipetting, and a single-cell suspension was achieved by forceful expulsion of the cells through a 22-gauge syringe needle. Total bone marrow cells (10^6^) were cultured in 36-cm^2^ Petri dishes in 5 mL of the medium described earlier, which was changed every 4 days, and cultures were maintained for 18 days. At the end of the culture period, cells were washed with PBS, fixed with paraformaldehyde/lysine/periodate fixative, and stained with methyl blue or cytochemically for alkaline phosphatase. After staining, the numbers of total colony-forming units fibroblastic (CFU-F) and ALP^+^ CFU-F were counted manually or by computer-assisted image analysis.

### MAPK assay

The second-passaged bone marrow mesenchymal stem cells from 2-week-old WT or *CaR*^–/–^ mice were cultured with DMEM containing 10% FCS, 50 µg/mL of ascorbic acid, 10 mM β-glycerophosphate, and 10^−8^ M dexamethasone until confluence and were transferred into serum-free DMEM/F12 calcium-deficient medium (Sigma) overnight. These cells then were treated with various reagents as described in the figure legends. After treatment, cells were washed in PBS (20 mM NaH_2_PO_4_, 0.9% NaCl, pH 7.4) and lysed for 20 minutes with lysis buffer (20 mM Tris-Cl, pH 7.4, 150 mM NaCl, 0.1% Nonidet P-40, 1% glycerol, 0.2 mM sodium vanadate, and a protease mixture tablet/10 mL of buffer). The samples were collected and microcentrifuged at 14,000 rpm for 5 minutes, and the supernatants were collected, assayed for protein, and prepared for Western blot analysis with antibody against the active phosphorylated form of mitogen-activated protein kinase (MAPK).(35) Membranes were stripped and reprobed with polyclonal antibodies against MAPK (ERK-1 and ERK-2).

### Western blot analysis

Proteins were extracted from the duodenum of 2-week-old WT or *CaR*^–/–^ mice and quantitated by a kit (Bio-Rad, Mississauga, Ontario, Canada). In total, 30 µg of protein samples was fractionated by SDS-PAGE and transferred to nitrocellulose membranes. Immunoblotting was carried out as described previously(36) using TRPV5 (ECaC1), calbindin-D_28K_, calbindin-D_9K_, and the Na^+^/Ca^2+^ exchanger (NCX1) (Swant, Bellinzona, Switzerland) and β-tubulin (Santa Cruz Biotechnology Inc., Santa Cruz, CA, USA). Bands were visualized using ECL chemiluminescence (Amersham, Piscataway, NJ, USA) and quantitated by Scion Image Beta 4.02 (Scion Corporation, Frederick, MD, USA).

### Computer-assisted image analysis

After H&E staining or histochemical or immunohistochemical staining of sections from six mice of each genotype, images of fields were photographed with a Sony (Tokyo, Japan) digital camera. Images of micrographs from single sections were digitally recorded using a rectangular template, and recordings were processed and analyzed using Northern Eclipse image-analysis software (Empix Imaging Inc.), as described previously.(32–34)

### Statistical analysis

Data from image analysis are presented as means ± SEM. Statistical comparisons were made using a two-way ANOVA, with *p* < .05 being considered significant.

## Results

### Effect of dietary calcium on milk calcium content and calcium-regulating hormones in milk

**Fig. 1:**
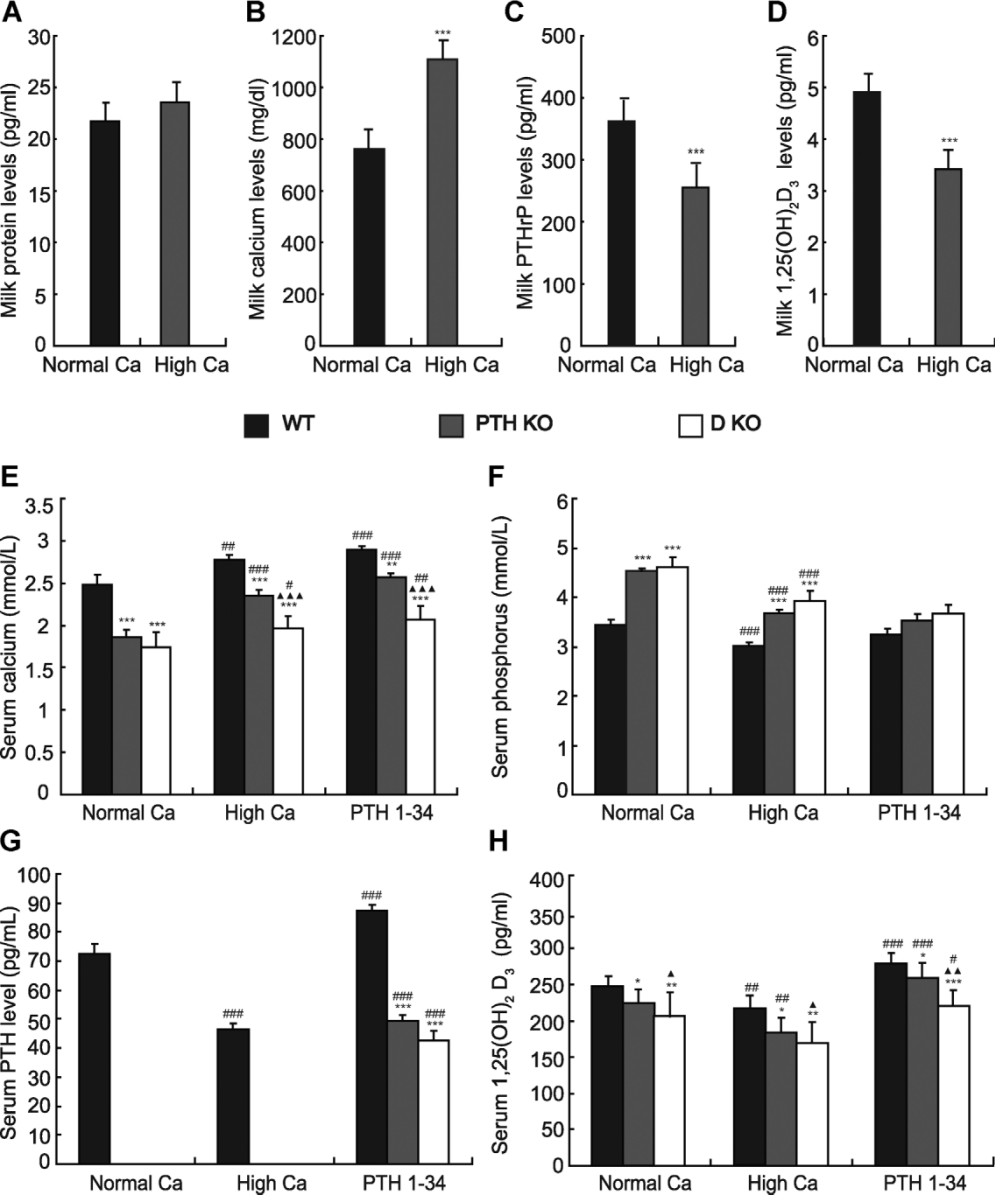
Effect of dietary calcium on milk calcium content and calcium-regulating hormones in dams and on serum calcium, phosphorus, PTH, and 1,25(OH)_2_D_3_ levels in *Pth*^–/–^ and *Pth*^–/–^*CaR*^–/–^ pups. (*A*) Milk calcium, (*B*) PTHrP, (*C*) 1,25(OH)_2_D_3_, and (*D*) protein concentrations were determined in the samples from *Pth*^+/–^*CaR*^+/–^ dams on day 14 of lactation on the normal diet (Normal Ca) or the high-calcium diet (High Ca) as described in “Materials and Methods.” (*E*) Serum calcium, (*F*) phosphorus, (*G*) PTH, and (*H*) 1,25(OH)_2_D_3_ levels were determined in samples from 3-week-old WT, *Pth*^–/–^ (*Pth* KO), and *Pth*^–/–^*CaR*^–/–^ (DKO) pups that were fed by dams on the normal diet or the high-calcium diet or were administered exogenous PTH(1–34). Each value is the mean ± SEM of determinations in 5 pups of each genotype. *p < .05; **p < .01; ***p < .001 compared with WT littermates of the same group; ^▴^*p* < .05; ^▴▴^*p* < .01; ^▴▴▴^*p* < .001 compared with *Pth*^–/–^ littermates of the same group; ^#^*p* < .05; ^##^*p* < .01; ^###^*p* < .001 compared with the genotype-matched pups fed by dams on the normal diet.

To determine whether dietary calcium affects milk calcium content and calcium-regulating hormones, milk calcium, 1,25(OH)_2_D_3_, and PTHrP levels were examined in lactating *Pth*^+/–^*CaR*^+/–^ dams fed either the normal diet or the high-calcium diet. The high-calcium diet increased milk calcium and decreased PTHrP and 1,25(OH)_2_D_3_ levels (Fig. 1*A–C*) but did not affect milk protein concentrations (Fig. 1*D*).

### Effects of dietary calcium and exogenous PTH on serum calcium, phosphorus, PTH, and 1,25(OH)_2_D_3_ in *Pth*^–/–^ and *Pth*^–/–^*CaR*^–/–^ pups

To determine whether maternal dietary calcium and exogenous PTH affect mineral homeostasis in the suckling pups, serum calcium, phosphorus, PTH, and 1,25(OH)_2_D_3_ levels were measured in 3-week-old pups fed by *Pth*^+/–^*CaR*^+/–^ dams on either the normal diet or the high-calcium diet or administrated exogenous PTH. Serum calcium levels were decreased in *Pth*^–/–^ and *Pth*^–/–^*CaR*^–/–^ pups fed by *Pth*^+/–^*CaR*^+/–^ dams on either the normal diet or the high-calcium diet or administrated exogenous PTH and were decreased more dramatically in *Pth*^–/–^*CaR*^–/–^ pups fed by *Pth*^+/–^*CaR*^+/–^ dams on the high-calcium diet or administrated exogenous PTH (Fig. 1*E*). Serum phosphorus levels were still elevated relative to WT mice in both mutants when fed by mothers on either the normal diet or the high-calcium diet and were reduced further in the genotype-matched pups fed by dams on the high-calcium diet compared with pups fed by dams on the normal diet (Fig. 1*F*). Serum phosphorus levels were normalized by exogenous PTH treatment of both mutant animals (Fig. 1*F*). Serum PTH levels in WT pups were decreased when the dams were on the high-calcium diet. Administration of PTH to pups increased PTH levels in WT pups fed by dams on the normal diet. Basal serum PTH levels in *Pth*^–/–^ and *Pth*^–/–^*CaR*^–/–^ pups were undetectable (Fig. 1*G*). Although exogenous PTH administration increased serum PTH levels in *Pth*^–/–^ and *Pth*^–/–^*CaR*^–/–^ pups, the levels were significantly lower than in their WT littermates (Fig. 1*G*). Serum 1,25(OH)_2_D_3_ levels were reduced in *Pth*^–/–^ pups and were even more reduced in *Pth*^–/–^*CaR*^–/–^ pups fed by dams on the normal diet. Feeding dams a high-calcium diet decreased serum 1,25(OH)_2_D_3_ levels significantly in WT and *Pth*^–/–^ pups but insignificantly in *Pth*^–/–^*CaR*^–/–^ pups compared with genotype-matched pups from dams fed the normal diet. PTH increased serum 1,25(OH)_2_D_3_ levels in all WT, *Pth*^–/–^, and *PTH*^–/–^*CaR*^–/–^ pups, but the percentage increase was less in *Pth*^–/–^*CaR*^–/–^ pups than in the other two genotypes (Fig. 1*H*).

### Effects of dietary calcium and exogenous PTH on bone growth, BMD, and bone volume in *Pth*^–/–^ and *Pth*^–/–^*CaR*^–/–^ pups

**Fig. 2:**
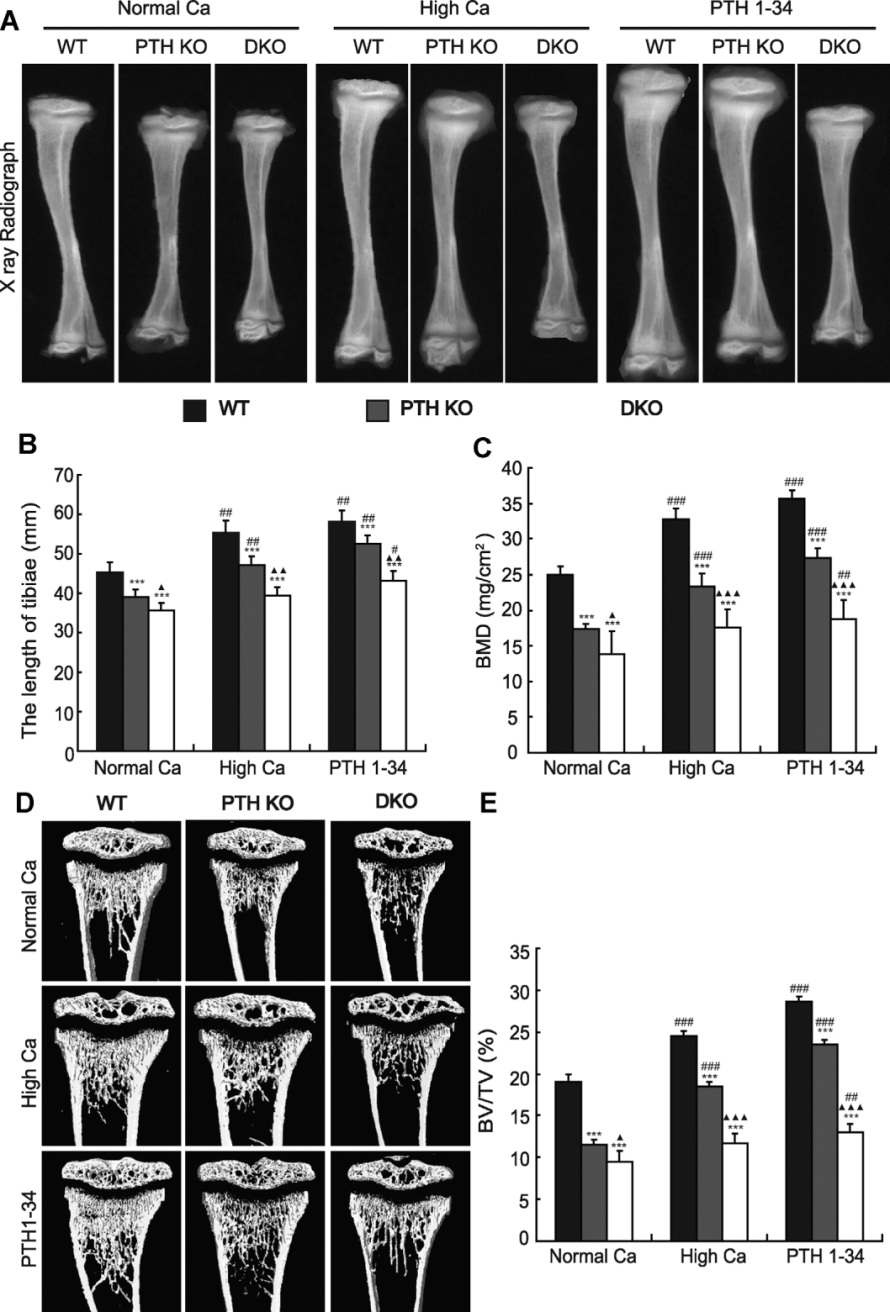
Effects of dietary calcium and exogenous PTH on bone growth, BMD, and bone volume in *Pth*^–/–^ and *Pth*^–/–^*CaR*^–/–^ pups. (*A*) X-rays of tibias, (*B*) the lengths of tibias, (*C*) BMD values, (*D*) representative longitudinal sections of the proximal ends of tibias by µCT and 3D reconstruction, and (*E*) trabecular bone volume (BV/TV) that were determined in the metaphyseal regions, including both primary and secondary spongiosa, of tibias from 3-week-old WT, *Pth*^–/–^, and *Pth*^–/–^*CaR*^–/–^ pups fed by dams on normal Ca or high Ca or were administered exogenous PTH(1–34). Each value is the mean ± SEM of determinations in 5 mice of each genotype. *p < .05; **p < .01; ***P < .001 compared with WT littermates of the same group; ^▴^*p* < .05; ^▴▴^*p* < .01; ^▴▴▴^*p* < .001 compared with *Pth*^–/–^ pups of the same group; ^#^*p* < .05; ^##^*p* < .01; ^###^*p* < .001 compared with the genotype-matched pups fed by dams on the normal diet.

To determine whether CaR deficiency influences the effects of dietary calcium and exogenous PTH on bone growth and BMD, 3-week-old pups were fed by *Pth*^+/–^*CaR*^+/–^ dams that received either the normal or the high-calcium diet, or pups were administrated exogenous PTH. Tibias then were examined by radiography (Fig. 2*A*), tibia lengths were measured (Fig. 2*B*), and BMD was determined using a PIXImus densitometer (Fig. 2*C*) in the 3-week-old pups.

*Pth*^–/–^ pups fed by dams receiving either a normal or a high-calcium diet had a significant reduction in bone length and BMD compared with WT pups. The reduction was more severe in *Pth*^–/–^*CaR*^–/–^ pups. Administering the high-calcium diet to dams increased the length and BMD in WT and *Pth*^–/–^ pups but not in *Pth*^–/–^*CaR*^–/–^ pups compared with those on the normal calcium diet. Two weeks of PTH treatment increased tibial length and BMD in all WT, *Pth*^–/–^, and *Pth*^–/–^*CaR*^–/–^ pups, but the percentage increase was less in *Pth*^–/–^*CaR*^–/–^ pups than in the other two genotypes. The percentage increases in bone length and BMD were greater in PTH-treated pups than in pups fed by dams on a high-calcium diet in both WT and *Pth*^–/–^ pups (Fig. 2*A–C*). To determine whether CaR deficiency modifies the effects of dietary calcium and exogenous PTH on bone volume, the trabecular bone volume was assessed by µCT (Fig. 2*D*) and histomorphometric analysis (Fig. 2*E*) in the proximal ends of tibias from mice treated as described earlier. Consistent with the changes in bone growth and BMD, *Pth*^–/–^ pups fed by dams on either the normal or the high-calcium diet had reduced trabecular bone volume compared with WT pups. Bone volume was decreased further in *Pth*^–/–^*CaR*^–/–^ pups. The bone volume was increased in WT and *Pth*^–/–^ pups but not in *Pth*^–/–^*CaR*^–/–^ pups fed by dams on a high-calcium diet compared with genotype-matched pups fed by dams on a normal calcium diet. PTH treatment increased the bone volume in all three types of pups, but the increase was smaller in *Pth*^–/–^*CaR*^–/–^ pups than in WT and *Pth*^–/–^ pups. The percentage increase in bone volume was higher in PTH-treated pups than in pups fed by dams on a high-calcium diet in both WT and *Pth*^–/–^ pups (Figs.2*D, E*).

### Effects of dietary calcium and exogenous PTH on osteoblastic bone formation in *Pth*^–/–^ and *Pth*^–/–^*CaR*^–/–^ pups

**Fig. 3:**
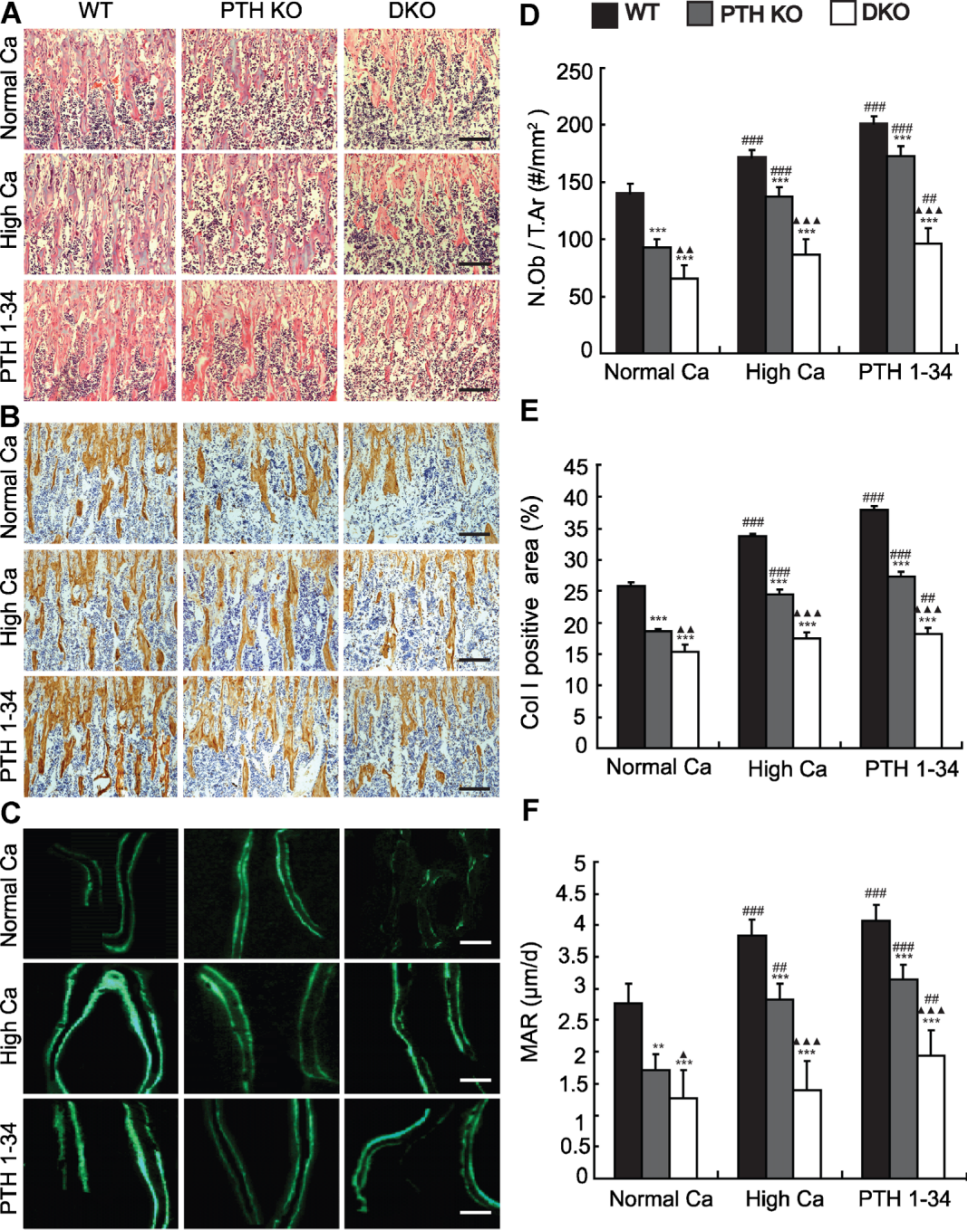
Effects of dietary calcium and exogenous PTH on osteoblastic bone formation in *Pth*^–/–^ and *Pth*^–/–^*CaR*^–/–^ pups. Representative micrographs of sections that were (*A*) stained with H&E, (*B*) immunostained for type 1 collagen (Col I), or (*C*) double calcein labeled from trabecular bone from 3-week-old WT, *Pth*^–/–^, and *Pth*^–/–^*CaR*^–/–^ pups fed by dams fed with normal Ca or high Ca or pups that were administered exogenous PTH(1–34). Scale bars in *A*, *B*, and *C* represent 50, 50, and 25 µm, respectively. (*D*) Numbers of osteoblasts per square millimeter (N.Ob/T.Ar, *n*/mm^2^) that were counted in the metaphyseal regions of H&E-stained tibial sectrions, (*E*) the type 1 collagen–positive area as a percentage of the tissue area was measured in the metaphyseal regions of tibial sections, and (*F*) MAR of trabecular bone. Each value is the mean ± SEM of determinations in 5 mice of each genotype. *p < .05; **p < .01; ***p < .001 compared with WT littermates of the same group; ^▴^*p* < .05; ^▴▴^*p* < .01; ^▴▴▴^*p* < .001 compared with *Pth*^–/–^ pups of the same group; ^#^*p* < .05; ^##^*p* < 0.01; ^###^*p* < .001 compared with the genotype-matched pups fed by dams on the normal diet.

**Fig. 4:**
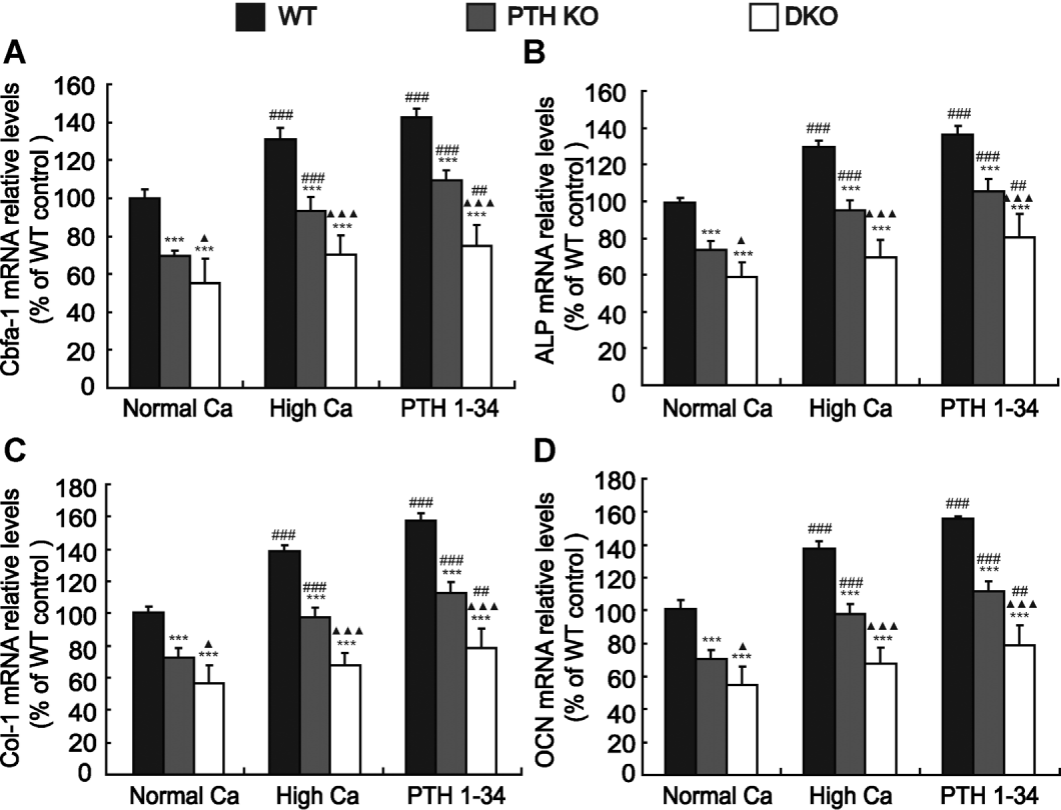
Effects of dietary calcium and exogenous PTH on the expression of osteoblastic genes in *Pth*^–/–^ and *Pth*^–/–^*CaR*^–/–^ pups. Real-time RT-PCR was performed on extracts of bone tissue from long bones of WT, *Pth*^–/–^, and *Pth*^–/–^*CaR*^–/–^ pups that were fed by dams on normal Ca or high Ca or were administered exogenous PTH(1–34) for the gene expression of (*A*) *Cbfa*-1,(*B*) *Alp*, (C) *type 1 collagen* (*ColI*), and (*D*) *osteocalcin* (*Ocn*) as described in “Materials and Methods.” Messenger RNA expression, assessed by real-time RT–PCR analysis, is calculated as a ratio to the *GAPDH* mRNA level and expressed relative to levels in WT mice. Each value is the mean ± SEM of determinations in 5 mice of each genotype. *p < .05; **p < .01; ***p < .001 compared with WT littermates of the same group; ^▴^*p* < .05; ^▴▴^*p* < .01; ^▴▴▴^*p* < .001 compared with *Pth*^–/–^ pups of the same group; ^#^*p* < .05; ^##^*p* < .01; ^###^*p* < .001 compared with the genotype-matched pups fed by dams on the normal diet.

To determine whether or not alterations of trabecular bone volume in *Pth*^–/–^ and *Pth*^–/–^*CaR*^–/–^ pups are associated with changes in osteoblast function, bone-formation parameters were assessed by staining with H&E (Fig. 3*A*), immunostaining for type 1 collagen (Fig. 3*B*), double calcein labeling (Fig. 3*C*) and histomorphometric analysis for osteoblast number (Fig. 3*D*), type 1 positive area (Fig. 3*E*), and mineral apposition rate (MAR; Fig. 3*F*). *Pth*^–/–^ pups fed by dams on the normal diet displayed a significantly reduced osteoblast number, type 1 collagen immunopositive area, and MAR compared with WT pups, and these parameters were decreased further in *Pth*^–/–^*CaR*^–/–^ pups. These bone-formation parameters were increased in WT and *Pth*^–/–^ pups but not in *Pth*^–/–^*CaR*^–/–^ pups fed by dams on the high-calcium diet compared with the pups fed by dams on the normal calcium diet. PTH treatment for 2 weeks increased bone formation in WT and *Pth*^–/–^ pups and *Pth*^–/–^*CaR*^–/–^ pups to a lesser extent. Exogenous PTH had a greater stimulatory effect on bone formation than in the pups fed by dams on the high-calcium diet in both WT and *Pth*^–/–^ pups (Fig. 3*A–F*). We also assessed whether these alterations of osteoblastic bone formation were associated with similar changes in the expression of several osteoblastic genes. The expression levels of *Cbfa-*1 (Fig. 4*A*), *Alp* (Fig. 4*B*), *type 1 collagen* (Fig. 4*C*), and *osteocalcin* (Fig. 4*D*) were examined by real-time RT-PCR. The alterations in the expression of transcripts for *Cbfa*-1, *Alp*, *type 1 collagen*, and *osteocalcin*, as assessed by real-time RT-PCR, were consistent with the alterations observed by histomorphometry.

### Effects of dietary calcium and exogenous PTH on osteoprogenitor commitment in *Pth*^–/–^ and *Pth*^–/–^*CaR*^–/–^ pups

**Fig. 5:**
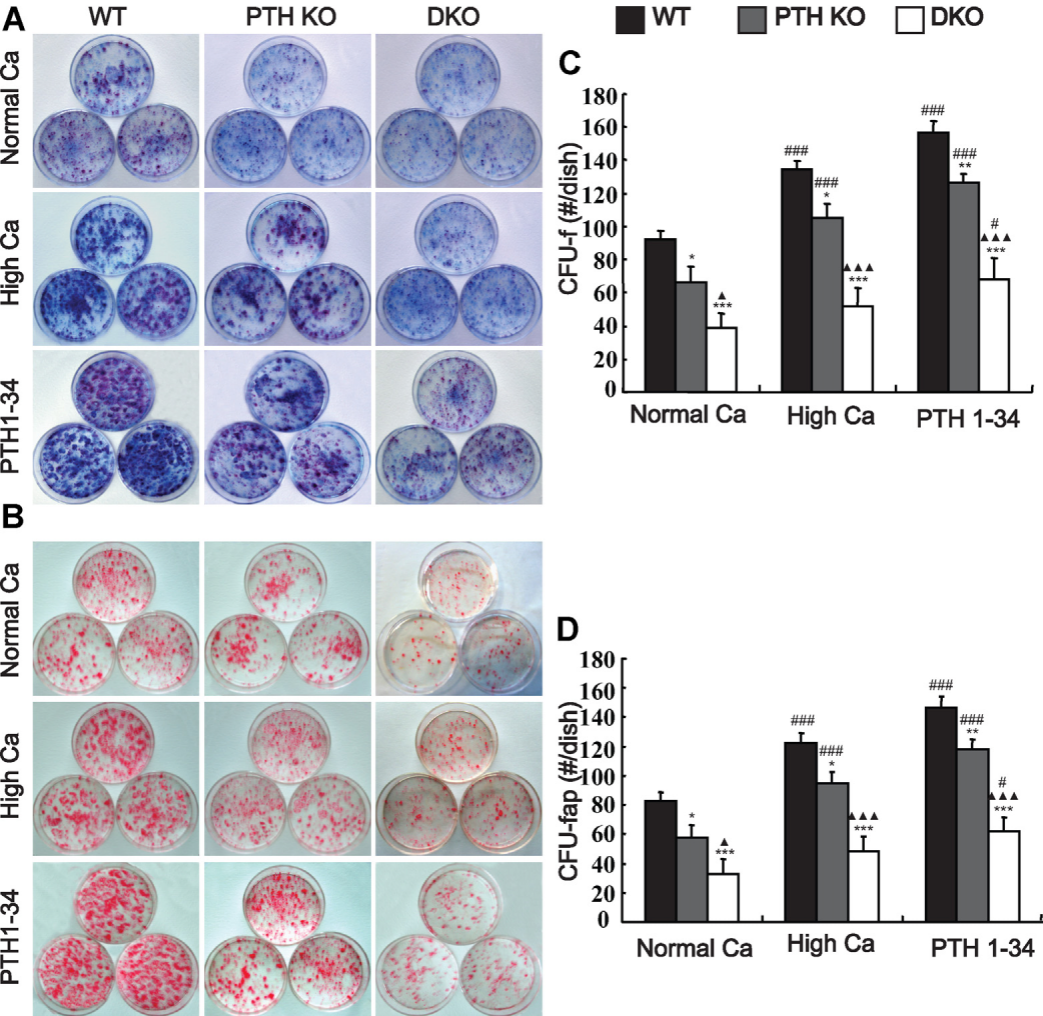
Effects of dietary calcium and exogenous PTH on osteoprogenitor commitment in *Pth*^–/–^ and *Pth*^–/–^*CaR*^–/–^ pups. Bone marrow cells from WT, *Pth*^–/–^, and *Pth*^–/–^*CaR*^–/–^ pups that were fed by dams on normal Ca or high Ca or were administered exogenous PTH(1–34) were cultured in osteogenic differentiation medium for 18 days and (*A*) stained with methylene blue for total number of colonies (CFU-F) or (*B*) cytochemically for ALP. (*C*) Total CFU-F number per dish. (*D*) ALP^+^ CFU-F (CFU-F_AP_) number per dish. Each value is the mean ± SEM of determinations in 5 mice of each genotype. *p < 0.05; **p < .01; ***p < .001 compared with WT littermates of the same group; ^▴^*p* < .05; ^▴▴^*p* < .01; ^▴▴▴^*p* < .001 compared with *Pth*^–/–^ pups of the same group; ^#^*p* < .05; ^##^*p* < .01; ^###^*p* < .001 compared with the genotype-matched pups fed by dams on the normal diet.

To investigate whether the reduction in osteoblast numbers in *Pth*^–/–^ and *Pth*^–/–^*CaR*^–/–^ pups is due to impaired commitment of bone marrow mesenchymal stem cells (MSCs) into osteogenic cells, colony-forming assays were performed using bone marrow stromal cells from these animals. Consistent with the bone-formation data, the total number of colony-forming units fibroblastic (CFU-F) and ALP^+^ CFU-F (CFU-F_AP_) in bones from *Pth*^–/–^ pups fed by dams on either the normal or the high-calcium diet were reduced significantly compared with WT pups, with a further decrease in *Pth*^–/–^*CaR*^–/–^ pups (Fig. 5). The high-calcium diet increased the numbers of CFU-F and CFU-F_ap_ in WT and *Pth*^–/–^ pups but not in *Pth*^–/–^*CaR*^–/–^ pups. Similar to the bone-formation parameters, PTH treatment increased CFU-F numbers in WT and *Pth*^–/–^ pups, with a lesser increase in *Pth*^–/–^*CaR*^–/–^ pups. The percentage increase in these osteogenic parameters was higher in PTH-treated pups than in pups fed by dams on the high-calcium diet in both WT and *Pth*^–/–^ pups (Fig. 5*A–D*).

### Effects of dietary calcium and exogenous PTH on osteoclastic bone resorption in *Pth*^–/–^ and *Pth*^–/–^*CaR*^–/–^ pups

**Fig. 6:**
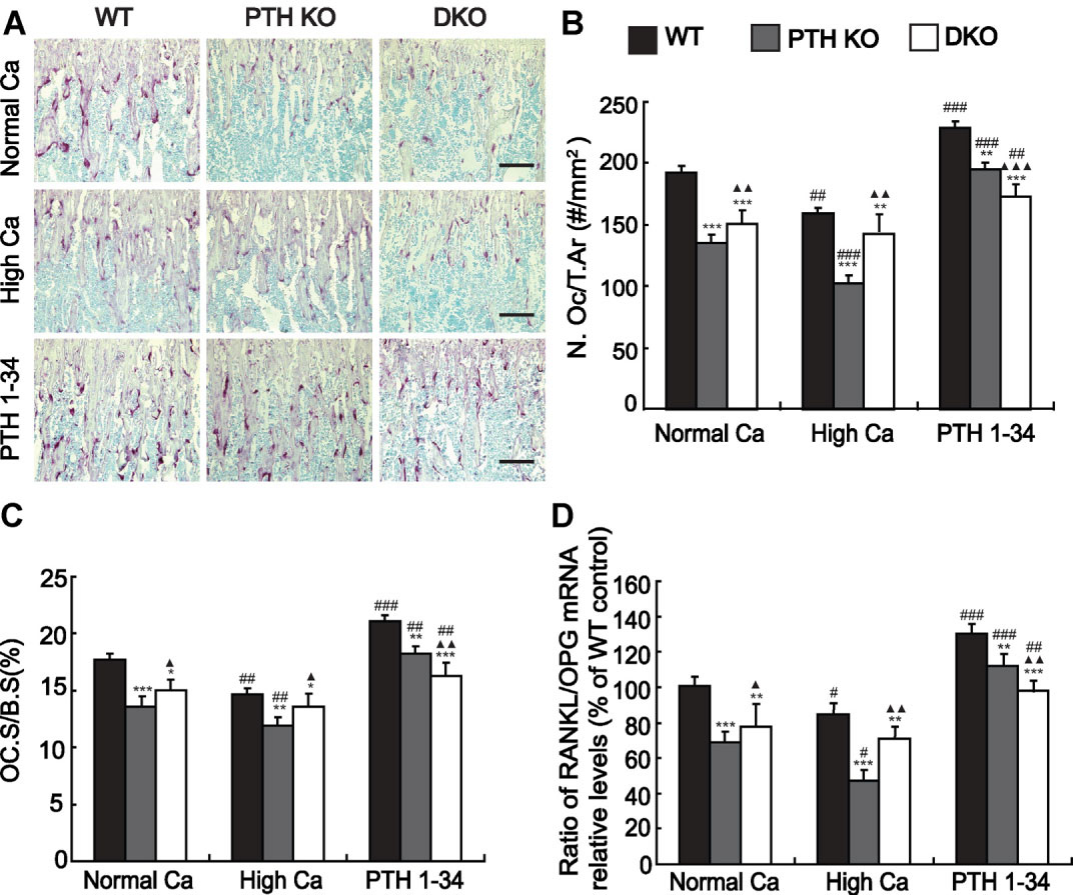
Effects of dietary calcium and exogenous PTH(1–34) on osteoclastic bone resorption in *Pth*^–/–^ and *Pth*^–/–^*CaR*^–/–^ pups. Representative micrographs of sections of the tibias from WT, *Pth*^–/–^, and *Pth*^–/–^*CaR*^–/–^ pups that were fed by dams on normal Ca or high Ca or were administered exogenous PTH(1–34) (*A*) stained histochemically for TRACP. Scale bar = 50 µm. (*B*) Number of TRACP^+^ osteoclasts related to tissue area (N.Oc/T.Ar, *n*/mm^2^) and (*C*) osteoclast surface relative to bone surface (Oc.S/BS, %) were counted in the metaphyseal regions of TRACP-stained tibial sections. (*D*) Real-time RT-PCR was performed on bone extracts for *Rankl* and *Opg* mRNA as described in “Materials and Methods.” Messenger RNA expression assessed by real-time RT-PCR analysis is calculated as a ratio to the *GAPDH* mRNA level and expressed relative to levels of WT mice. Ratio of *Rankl*/*Opg* relative mRNA levels was calculated. Each value is the mean ± SEM of determinations in 5 mice of each group. *p < .05; **p < .01; ***p < .001 compared with WT littermates of the same group; ^▴^*p* < .05; ^▴▴^*p* < .01; ^▴▴▴^*p* < .001 compared with *Pth*^–/–^ pups of the same group; ^#^*p* < .05; ^##^*p* < .01; ^###^*p* < .001 compared with the genotype-matched pups fed by dams on the normal diet.

To determine whether alterations in osteoclast function also contribute to decreased bone volume in *Pth*^–/–^ and *Pth*^–/–^*CaR*^–/–^ pups, osteoclast number and surface were determined by histomorphometric analysis on TRACP-stained sections (Fig. 6). We also examined the expression levels of *Rankl* and *Opg* mRNA in bony tissues by real-time RT-PCR and calculated the ratio of *Rankl/Opg*. The number and surfaces of TRACP^+^ osteoclasts and the ratio of *Rankl/Opg* were reduced significantly in *Pth*^–/–^ pups and less significantly in *Pth*^–/–^*CaR*^–/–^ pups compared with WT pups fed by dams on either the normal or the high-calcium diet. These parameters were reduced significantly in WT and *Pth*^–/–^ pups but did not decrease significantly in *Pth*^–/–^*CaR*^–/–^ pups fed by dams on the high-calcium diet compared with genotype-matched pups fed by dams on the normal diet. These parameters increased significantly in PTH-treated WT and *Pth*^–/–^ pups and less significantly in PTH-treated *Pth*^–/–^*CaR*^–/–^ pups compared with vehicle-treated genotype-matched pups fed by dams on the normal diet (Fig. 6*A–D*).

### Effect of CaR deficiency on osteoprogenitor intracellular signaling, intestinal calcium transporter expression, and urinary calcium excretion

**Fig. 7:**
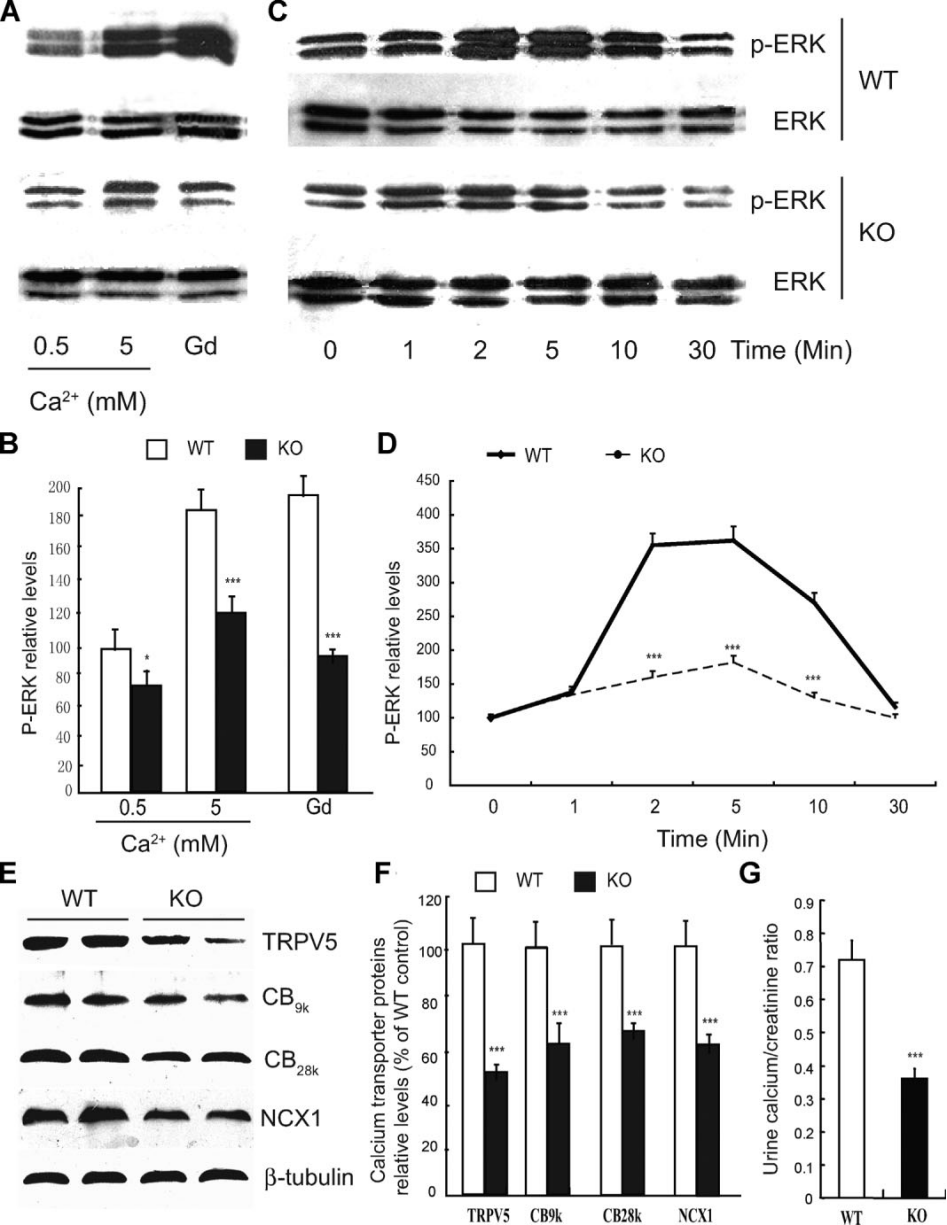
Effect of CaR deficiency on osteoprogenitor intracellular signaling, intestinal calcium transporter expression, and renal calcium excretion. (*A*) Second-passaged bone marrow MSCs from WT and *CaR*^–/–^ (KO) mice were treated with 0.5 and 5 mM CaCl_2_ or 50 µM Gd^3+^ for 5 minutes, and cells were analyzed by Western blot for ERK phosphorylation activation (p-ERK) and total ERK (ERK). (*B*) p-ERK level relative to total ERK level was assessed by densitometric analysis and expressed relative to levels of cells from WT mice treated with 0.5 mM CaCl_2_. (*C*) The cells were treated with 5 mM CaCl_2_ for various times and were analyzed by Western blot for ERK phosphorylation activation (p-ERK) and total ERK (ERK). (*D*) p-ERK level relative to total ERK level was assessed by densitometric analysis and expressed relative to levels of cells from untreated cultures. (*E*) Western blots of extracts of duodenums from WT and *CaR*^–/–^ (KO) mice for expression of TRPV5, calbindin-D_28K_ (CB28K), calbindin-D_9K_ (CB9K), and NCX1. β-Tubulin was used as a loading control for Western blots. (*F*) TRPV5, CB28K, CB9K and NCX protein levels relative to β-tubulin protein level were assessed by densitometric analysis and expressed relative to levels of WT mice. (*G*) Urine calcium and urine creatinine were measured as described in “Materials and Methods,” and the urine calcium/creatinine ratio was calculated. *p < .05; **p < .01; ***p < .001 compared with WT mice.

To determine whether bone marrow MSCs from CaR-deficient mice respond to signaling changes in extracellular calcium (Ca^2+^) concentrations, cells were treated with either a high concentration of Ca^2+^ or with the nonpermeant CaR agonist gadolinium (Gd^3+^), and ERK1/2 phosphorylation was determined by Western blots with an antibody against the active, phosphorylated form of ERK1/2. Basal levels of ERK1/2 in the presence of 0.5 mM Ca^2+^ were significantly lower in cells from *CaR*^–/–^ mice than in cells from WT mice. Elevated Ca^2+^ or Gd^3+^ treatment significantly induced ERK1/2 activation in the bone marrow MSCs from both WT and *CaR*^–/–^ mice at 5 minutes. However, the high-Ca^2+^-induced ERK1/2 activation was markedly more pronounced in bone marrow MSCs from WT mice than from *CaR*^–/–^ mice. The Gd^3+^-induced ERK1/2 activation in bone marrow MSCs from WT mice was even more pronounced than in cells from *CaR*^–/–^ mice, where Gd^3+^-stimulated ERK1/2 activation barely reached the basal levels of the WT mice (Fig. 7*A, B*). High-Ca^2+^-induced ERK1/2 activation was time-dependent in the cells from both WT and *CaR*^–/–^ mice. However, ERK1/2 activation from 2 to 10 minutes was more markedly stimulated by 5 mM Ca^2+^ in cells from WT mice than in cells from *CaR*^–/–^ mice (Fig. 7*C, D*).

To determine whether the ability of Ca^2+^ transport in the duodenum is altered in the CaR-deficient mice, the expression of intestinal calcium transporters was examined in duodenums from WT and *CaR*^–/–^ mice by Western blots. The results revealed that protein expression levels of TRPV5, calbindin-D_28K_, calbindin-D_9K_, and NCX1 in the duodenum were reduced in *CaR*^–/–^ mice compared with their WT littermates (Fig. 7*E, F*). When urine calcium and creatinine levels were measured and the urine calcium/creatinine ratio was calculated, we found that the urine calcium/creatinine ratio was lower in *CaR*^–/–^ mice than in their WT littermates (Fig. 7*G*).

## Discussion

We employed a genetic approach to determine whether deficiency of PTH alone or in combination with loss of CaR leads to alterations in calcium and skeletal homeostasis in neonatal animals in vivo, and we investigated the involvement of dietary calcium supplementation or exogenous PTH administration in these processes using *Pth*^–/–^ and *Pth*^–/–^*CaR*^–/–^ pups fed by dams on a normal or a high-calcium diet. We demonstrated that deletion of both PTH and CaR in suckling neonates leads to more severe defects in skeletal development and osteoblastic bone formation than in pups with deletion of PTH alone. Thus the length of long bones; BMD values; trabecular volume; osteoblast number; type 1 collagen deposition in the bone matrix; mineral apposition rate; and gene expression of *Cbfa*-1, *Alp*, *type 1 collagen*, and *osteocalcin*, as well as CFU-F forming efficiency from ex vivo bone marrow cultures, all were diminished more dramatically in *Pth*^–/–^*CaR*^–/–^ pups than *Pth*^–/–^ pups. Thus ablation of exon 5 of the *CaR* gene, encoding a portion of the extracellular domain of CaR, results in impairment in skeletal development and osteoblastic bone formation, suggesting that the CaR is essential for normal neonatal bone development and bone formation in vivo. A previous study did not find any obvious abnormalities in the cartilage and bone of adult *Pth*^–/–^*CaR*^–/–^ mice,(16) and it has been suggested that an alternatively spliced form of the CaR transcript, lacking exon 5, may compensate for loss of the full-length CaR in adult mice with knockout of exon 5 of the receptor. This alternatively spliced CaR transcript is generated in the growth plate,(37) skin,(38) and kidney(38) of these mice and potentially could explain these earlier observations.(16,39) However, whether this is a partial or full compensation has not been established. Our results demonstrate that this compensation is not full, at least under the conditions studied here, which differ greatly, however, from those in the prior studies. The very modest ERK1/2 activation by extracellular calcium in bone marrow MSCs of *CaR*^–/–^ mice is consistent with at most a partially responsive CaR in skeletal cells, and the decreased urine calcium excretion found in the *CaR*^–/–^ mice is consistent with the loss of renal function of the CaR. Nevertheless, although the skeletal defects observed in our mouse models appeared to be mainly due to the deficiency of CaR function in bone-related cells, defective CaR functions in the kidney and possibly in the gut also may contribute significantly to the development of the bone phenotype in vivo.

We further investigated whether CaR deficiency affects skeletal responses to dietary calcium supplementation. Because the calcium source for suckling neonates is maternal milk, we altered the dietary content of the dams to improve calcium intake in the neonates. Our results show that a high-calcium diet increases the calcium levels in milk of the lactating mothers. In contrast, milk PTHrP and 1,25(OH)_2_D_3_ levels were reduced in dams fed the high-calcium diet compared with dams fed the normal calcium diet. Previous study has shown that the CaR stimulates calcium transport into milk and inhibits PTHrP secretion by the breast.(40) In view of the fact that the protein levels in milk were not substantially different, dietary calcium appeared to influence milk calcium independent of changes in protein. We also demonstrate that serum calcium, phosphorus, PTH, and 1,25(OH)_2_D_3_ levels are altered in sucking pups by altering the calcium in the diet of the lactating mothers. These results suggest that the high-calcium diet can elevate milk calcium significantly in lactating mothers, which subsequently results in changes of serum ion homeostasis and calcitrophic hormones in suckling neonates. These findings are consistent with our previous report related to the effect of dietary calcium supplementation on milk calcium content and calcium-regulating hormones in dams and on serum calcium, phosphorus, and 1,25(OH)_2_D_3_ in suckling pups.(28)

We found that the loss of CaR in *Pth*^–/–^*CaR*^–/–^ pups abolished dietary calcium supplement–mediated augmentation of osteoblastic bone formation. Thus, compared with genotype-matched pups fed by dams on the normal diet, skeletal growth and bone-formation parameters were increased in the WT and *Pth*^–/–^ pups but not in *Pth*^–/–^*CaR*^–/–^ pups fed by dams on the high-calcium diet. These results, demonstrating that CaR deficiency in vivo abolishes skeletal responses to dietary calcium supplementation in suckling neonates, are consistent with previously published in vitro findings in which exposure of primary osteoblasts or a variety of osteoblast-like cells to high calcium or the polycationic CaR agonists neomycin and gadolinium stimulates their proliferation, differentiation, and mineralization by activating p42/44 MAPK, p38 MAPK, and/or JNK pathways.(5,7,41,42) Although an alternate skeletal calcium receptor, GPRC6A, has been described(43,44) through which calcium might act, its role in skeletal physiology is unclear because conflicting reports on the skeletal phenotype of *Gprc6a* null mice have been reported,(44,45) and no calcium channel or transporter in bone has been reported consistently to alter osteoblast and osteoclast activity in response to changes in serum calcium. Consequently, the calcium effect appears to be via CaR. Although serum calcium increases were more modest in the *Pth*^–/–^*CaR*^–/–^ mice than in the WT and *Pth*^–/–^ mice, our conclusion would be that even if serum calcium were elevated to the same degree in all phenotypes we employed in this study, the absence of the CaR would be the factor reducing skeletal responsiveness. Our results therefore strongly indicate that CaR plays an important role in mediating dietary calcium-induced changes in both bone formation and resorption.in neonates in vivo.

We demonstrated previously that the skeletal anabolic action of PTH in neonates results not only from its direct action via the PTH receptor to increase the osteoblast pool but also by an indirect action mediated through increasing extracellular calcium concentration.(28) In this study, we further demonstrated that increasing the extracellular calcium concentration acts cooperatively with PTH to promote osteoblastic bone formation in the neonate and that the effect of the former is mediated via the CaR. When 1-week-old WT, *Pth*^–/–^, and *Pth*^–/–^*CaR*^–/–^ pups were injected with PTH(1–34) daily for 2 weeks, PTH(1–34) administration raised serum calcium and 1,25(OH)_2_D_3_ levels and reduced serum phosphorus levels significantly in WT and *Pth*^–/–^ pups and less significantly in *Pth*^–/–^*CaR*^–/–^ pups. The length of long bones; BMD values; trabecular volume; osteoblast number; type 1 collagen deposition in bone matrix; MAR; and the gene expression of *Cbfa*-1, *Alp*, *type 1 collagen*, and *osteocalcin*, as well as CFU-F forming potential from ex vivo bone marrow cultures, all were increased significantly in WT and *Pth*^–/–^ pups and less significantly in *Pth*^–/–^*CaR*^–/–^ pups. Thus it is likely that the CaR is required for an optimal skeletal anabolic action of PTH in neonates.

Previous studies have revealed the importance of regulation of osteoclast function by extracellular calcium. Exposing osteoclasts to very high calcium, as seen only in the bone microenvironment, results in dramatic cell retraction followed by a profound inhibition of bone resorption.(46–49) It has been shown that such very high calcium levels inhibit bone-resorbing activity of osteoclasts by directly acting on the CaR, which is expressed by osteoclast precursors(6) and mature osteoclasts.(10,11) Furthermore, mature osteoclasts undergo apoptosis in the presence of very high calcium, and a dominant-negative CaR construct abrogates this effect, indicating the role of CaR in osteoclast apoptosis. The signaling pathways that are associated with induction of osteoclast apoptosis by the CaR likely involve PLC and NF-κB. Evidence also has been provided recently to support the role of CaR in osteoclast differentiation, and using *CaR*^–/–^ mice, Mentaverri and colleagues(11) have shown that osteoclast differentiation from bone marrow precursor cells in vitro is 70% less in *CaR*^–/–^ mice compared with WT mice. Taken together, these studies suggest that CaR activation is linked to physiologic responses of both enhanced differentiation and inhibition of bone-resorbing activity, although it is not clear if the calcium concentrations required for these processes are equivalent. In this study, we found that TRACP^+^ osteoclast numbers and surfaces, as well as the ratio of *Rankl/OPG*, were reduced more dramatically in *Pth*^–/–^ pups than in *Pth*^–/–^*CaR*^–/–^ pups fed by dams on the normal diet and, unlike pups from dams fed a high-calcium intake, were not further reduced significantly in *Pth*^–/–^*CaR*^–/–^ pups fed by dams on the same diet. Taken together, our observations suggest that in the model used, CaR is needed to support the calcemic action of PTH and to mediate calcium-induced inhibition of osteoclastic bone resorption, as suggested by earlier studies.(11)

Our findings are relevant to neonates but may differ in adult CaR-deficient mice. Thus our previous studies had shown that the bone phenotypes of *Pth*^–/–^ mice are different before and after weaning. Trabecular bone volume was reduced significantly in newborn and 2- and 3-week-old *Pth*^–/–^ pups compared with their WT littermates.(28,29,36) In contrast, trabecular bone volume was increased significantly in 4-month-old *Pth*^–/–^ mice compared with their WT littermates.(32,34) Although it is unclear why PTH deficiency results in such different bone phenotypes before weaning and in adulthood, such changes may be related to the different forms of food intake in these two settings. Mice are breast-fed before weaning but eat mouse chow thereafter. Breast milk not only contains a higher calcium concentration than the normal diet fed after weaning but also contains low levels of 1,25(OH)_2_D_3_,(28) both of which will have an impact on intestinal calcium absorption. Physiologic adaptation to these environmental changes may involve alterations in the function and/or efficacy of calcium-regulating hormones, including PTH, vitamin D, and calcium.

In summary, this study describes the role of the CaR in skeletal turnover in neonates and for the first time demonstrates that in the neonate, the CaR is essential to mediate changes in bone turnover resulting from alterations in dietary calcium as well as to facilitate the increased bone turnover elicited by PTH.
